# Short-term effects of intravenous batroxobin in treatment of sudden sensorineural hearing loss: a propensity score-matched study

**DOI:** 10.3389/fneur.2023.1102297

**Published:** 2023-04-17

**Authors:** Mengzhu Jiang, Huping Huang, Lingyun Mei, Chufeng He, Xinzhang Cai, Lu Jiang, Hong Wu, Xin Wang, Xuewen Wu

**Affiliations:** ^1^Department of Otolaryngology Head and Neck Surgery, Xiangya Hospital, Central South University, Changsha, Hunan, China; ^2^Province Key Laboratory of Otolaryngology Critical Diseases, Changsha, Hunan, China; ^3^National Clinical Research Center for Geriatric Disorders, Xiangya Hospital, Central South University, Changsha, China

**Keywords:** intravenous batroxobin, sudden sensorineural hearing loss, short-term effects, propensity score matching, treatment

## Abstract

**Background:**

Sudden sensorineural hearing loss (SSNHL) can cause great panic in patients. Whether it is advantageous to add intravenous batroxobin in the treatment of SSNHL remains to be determined. This study aimed to compare the short-term efficacy of therapy combined with intravenous batroxobin and that without intravenous batroxobin in SSNHL patients.

**Methods:**

This retrospective study harvested the data of SSNHL patients hospitalized in our department from January 2008 to April 2021. The hearing levels on the admitted day (before treatment) and the discharge day were considered pre-treatment hearing and post-treatment hearing, respectively. The hearing gain was the difference value of pre-treatment hearing and post-treatment hearing. We used Siegel's criteria and the Chinese Medical Association of Otolaryngology (CMAO) criteria to evaluate hearing recovery. The complete recovery rate, overall effective rate, and hearing gain at each frequency were considered outcomes. Propensity score matching (PSM) was conducted to balance the baseline characteristics between the batroxobin group and the non-batroxobin group. Sensitivity analysis was carried out in flat-type and total-deafness SSNHL patients.

**Results:**

During the study period, 657 patients with SSNHL were admitted to our department. Among them, a total of 274 patients met the enrolled criteria of our study. After PSM, 162 patients (81 in each group) were included in the analysis. Once the hospitalized treatment was completed, the patients would be discharged the next day. Logistic regression analysis of the propensity score-matched cohort indicated that both the complete recovery rates [Siegel's criteria, OR: 0.734, 95% CI: 0.368–1.466, *p* = 0.381; CMAO criteria, OR: 0.879, 95% CI: 0.435–1.777, *p* = 0.720] and the overall effective rates [Siegel's criteria and CMAO criteria, OR: 0.741, 95% CI: 0.399–1.378, *p* = 0.344] were not significantly different between the two treatment groups. Sensitivity analysis has shown similar results. For flat-type and total-deafness SSNHL patients, no significant difference was found in post-treatment hearing gain at each frequency between the two groups after PSM.

**Conclusion:**

There was no significant difference in short-term hearing outcomes between treatment with batroxobin and treatment without batroxobin in SSNHL patients by Siegel's and CMAO criteria after PSM. Future studies for better therapy regimens of SSNHL are still needed.

## Introduction

Sudden sensorineural hearing loss (SSNHL) can cause great panic in patients. It is often accompanied by tinnitus and/or vertigo and can have severe influences on the living quality of patients. Approximately 5–27 per 100,000 people in the United States suffer from SSNHL each year (1). In Germany, SSNHL affects 160–400/100,000 people per year (2). However, the etiology of SSNHL is still unclear. Viral infections, circulatory disorders, autoimmune diseases, and inner ear or central nervous system abnormalities have been proposed to be possible causative factors (3). Among them, inner ear microcirculation obstruction resulting from circulatory factors is a frequently theorized cause (3–5).

Aimed at the possible inner ear microcirculation ischemia, one may assume the application of defibrinogenation agents in the treatment of SSNHL. However, defibrinogenation therapy for SSNHL is still controversial. Batroxobin is a kind of defibrinogenation drug from the venom of *Bothrops atrox*, which cleaves plasma fibrinogen into fibrin (6). It can augment local blood flow by reducing blood viscosity (7, 8), and thereby improve microcirculation. Batroxobin was originally used in the treatment of occlusive vascular diseases, such as stroke and myocardial infarction (9, 10). Some studies indicated that intravenous batroxobin may be effective in treating patients with SSNHL (8, 11). In the German Guideline “Sudden Idiopathic Sensorineural Hearing Loss” (2011) (2), the reduction of fibrinogen was presented as rheological therapy, one of the two main methods for the treatment of SSNHL. In the Chinese Guideline for diagnosis and treatment of sudden deafness (2015), intravenous batroxobin was recommended in the treatment of flat-type and total-deafness SSNHL (12). A study in Japan has shown that the recovery rate of profound hearing loss patients was better in batroxobin therapy compared to that in steroid treatment (7). Meanwhile, Jia et al. (13) also proposed that patients with SSNHL receiving combined treatment (steroid and intravenous batroxobin) could achieve superior recovery than steroid therapy alone. However, based on the Clinical Practice Guideline: Sudden Hearing Loss of the American Academy of Otolaryngology-Head and Neck Surgery Foundation (AAO-HNSF) (2019) (1), clinicians should not routinely apply defibrinogen therapy to SSNHL patients. There was not enough evidence to conclude the benefit of defibrinogenation therapy in the management of SSNHL. Whether it is advantageous to add intravenous batroxobin in the treatment of SSNHL remains to be determined.

This retrospective propensity score-matched study aimed to compare the short-term efficacy of therapy combined with intravenous batroxobin and that without intravenous batroxobin in SSNHL patients. We attempted to explore whether it is better to add intravenous batroxobin in the therapy of SSNHL and to provide a new direction and thinking for the treatment of SSNHL.

## Materials and methods

### SSNHL patients

The study harvested the data of SSNHL patients hospitalized in our department from January 2008 to April 2021. In this study, SSNHL referred to a rapid onset sensorineural hearing loss that occurs within a 72-h window with a decrease in the hearing of ≥30 decibels (dB) affecting at least three consecutive frequencies (1). Pure-tone audiometry was conducted on the admitted day (before treatment). Hearing thresholds at 250, 500, 1,000, 2,000, 4,000, and 8,000 Hz were recorded. Hearing loss was defined concerning the opposite ear's thresholds. The pre-treatment audiograms were categorized into four types (12): (1) low-frequency: the hearing loss was at frequencies below 1,000 Hz (1,000 Hz included), at least hearing loss at 250 and 500 Hz was ≥20 dB HL; (2) high-frequency: the hearing loss was at frequencies above 2,000 Hz (2,000 Hz included), at least hearing loss at 4,000 and 8,000 Hz was ≥20 dB; (3) flat: hearing decreases at all frequencies with the average hearing threshold at 250, 500, 1,000, 2,000, 4,000, and 8,000 Hz ≤ 80 dB; (4) total-deafness: hearing decreases at all frequencies with the average hearing threshold at 250, 500, 1,000, 2,000, 4,000, and 8,000 Hz ≥81 dB. All patients must meet the inclusion standards: (1) unilateral SSNHL; (2) age ≥18 years old; (3) time from the onset of hearing loss to the start of treatment ≤ 14 days; (4) first treatment in our department; (5) hospitalized for SSNHL treatment; (6) no acoustic neuroma or other retro-cochlear lesions from the magnetic resonance imaging of the internal acoustic canal; (7) no history of otitis media, Meniere's disease, autoimmune diseases, long-term exposure to noise, use of ototoxic drugs, previous ear surgery, and prior sudden deafness or trauma; (8) no history of familial deafness; (9) no contraindications of drugs here; and (10) with complete medical records. Pregnant women were excluded. The study was approved by the ethical committee of Xiangya Hospital, Central South University (No. 2019051092). All the patients were de-identified when collecting information. Given the retrospective and de-identified nature of this study, the committee waived the obligation to obtain informed consent. The study was performed in accordance with the STROBE guidelines.

### Therapeutic regimens of intravenous batroxobin

The treatment regimen for each patient was formulated by sophisticated otolaryngologists in our department. Treatment including intravenous batroxobin was deemed as therapy combined with batroxobin (the batroxobin group). All other therapies were classified as therapy without batroxobin (the non-batroxobin group). No patient included in this study was treated with intravenous batroxobin alone. Batroxobin was administered intravenously once every other day for a total of three times. The initial dose was 10 BU and then decreased to a dose of 5 BU for the latter two times. The duration of each infusion was no <1 h. Serum fibrinogen was examined 24 h after each administration. If the level of serum fibrinogen was less than 100 mg/dl, it should be measured again after 24 h. The administration can carry on only when the fibrinogen level was higher than 100 mg/dl. In case complete recovery of hearing loss was confirmed by a pure-tone average (PTA), the treatment was interrupted.

### Measurement of outcomes

The pure-tone audiometry was performed again on the discharge day. The hearing levels on the admitted day (before treatment) and the discharge day were considered the pre-treatment hearing and post-treatment hearing, respectively. The hearing gain was the difference value of pre-treatment hearing and post-treatment hearing. We used Siegel's criteria to evaluate hearing recovery (14): (1) no improvement: the hearing gain is <15 dB; (2) slight improvement: final hearing level was poorer than 45 dB with a hearing gain over 15 dB; (3) partial recovery: final hearing level was from 25 to 45 dB with a hearing gain more than 15 dB; and (4) complete recovery: final hearing level was better than 25 dB. All comparison was made using the average thresholds at 500, 1,000, and 2,000 Hz (14). We also used the Chinese Medical Association of Otolaryngology (CMAO) criteria (12) when grading hearing recovery: (1) complete recovery: the hearing thresholds at the influenced frequencies return to the normal level (within 20 dB), or the same hearing level of the contralateral ear; (2) excellent recovery: the average of hearing gains at the influenced frequencies is ≥30 dB; (3) partial recovery: the average of hearing gains at the influenced frequencies is ≥15 dB but <30 dB; and (4) no recovery: the average of hearing gains at the influenced frequencies is <15 dB. As for flat-type and total-deafness SSNHL patients, the calculation involved hearing thresholds at all frequencies. Overall, effective rates (including complete recovery, partial recovery, and a slight improvement for Siegel's criteria; including complete recovery, excellent recovery, and partial recovery for the CMAO criteria) were calculated. Hearing gain at each frequency for flat-type and total-deafness patients was recorded as well.

### Potential confounders

The following data were collected as covariates: age, gender, course of the disease, type of the pre-treatment audiogram, history of hypertension and diabetes, pre-treatment platelet and fibrinogen level, use of steroids, and use of hyperbaric oxygen therapy (HBOT). The use of steroids was categorized as systemic corticosteroids alone, intratympanic corticosteroids alone, and together. Systemic corticosteroids were prednisone (≤ 60 mg/day, oral) or dexamethasone (10 mg/day, intravenous administration), full dose for 5 days and then halved, with a total duration of not more than 10 days. Intratympanic corticosteroids were dexamethasone (5 mg), which was injected every other day, three to five times in total. HBOT was conducted routinely twice a day, normally 10–14 times in total, with no more than 20 times.

### Statistical analysis

Continuous variables were described as mean with standard deviation (SD) for normally distributed variables and median with an interquartile range (IQR) for non-normally distributed variables. Categorical variables were shown as numbers and percentages. Propensity score matching (PSM) was performed as an adjustment of possible confounders between patients treated combined with batroxobin and patients treated without batroxobin. The matching was performed in a 1:1 ratio without replacement and with a caliper of 0.1. The scores were calculated and matched using nearest neighbor matching. All covariates were included in the propensity score model, and a logistic regression analysis was performed to calculate the propensity score as a logistic model. The absolute value of the standardized difference (ASD) was used to evaluate the balance between the batroxobin group and the non-batroxobin group before and after PSM. This value was set at <0.1 to balance the two groups. We also use the distribution of propensity scores of the two groups to reflect whether the two groups are balanced. The more similar the patterns are between the treated group and the control group, the better these two groups are balanced. After confirming that the two groups were well-balanced, logistic regression analyses were performed for the complete recovery rate and overall effective rate in both the entire cohort and the propensity score-matched cohort. Subsequently, sensitivity analysis was conducted as follows: the abovementioned matched analysis using propensity scores, applying the same covariates and matching algorithm detailed earlier, was performed in flat-type and total-deafness SSNHL patients. We also conducted a paired *t*-test to compare hearing gain at each frequency in flat-type and total-deafness SSNHL patients between the batroxobin and non-batroxobin groups. A *p*-value of <0.05 was considered to be statistically significant. SPSS (version 23.0, IBM) and R (version 3.1.0, R Foundation for Statistical Computing) were utilized for statistical analysis. GraphPad Prism (version 8.0.2, GraphPad Software) was used for drawing bar charts.

## Results

### SSNHL patients

**Figure 1:**
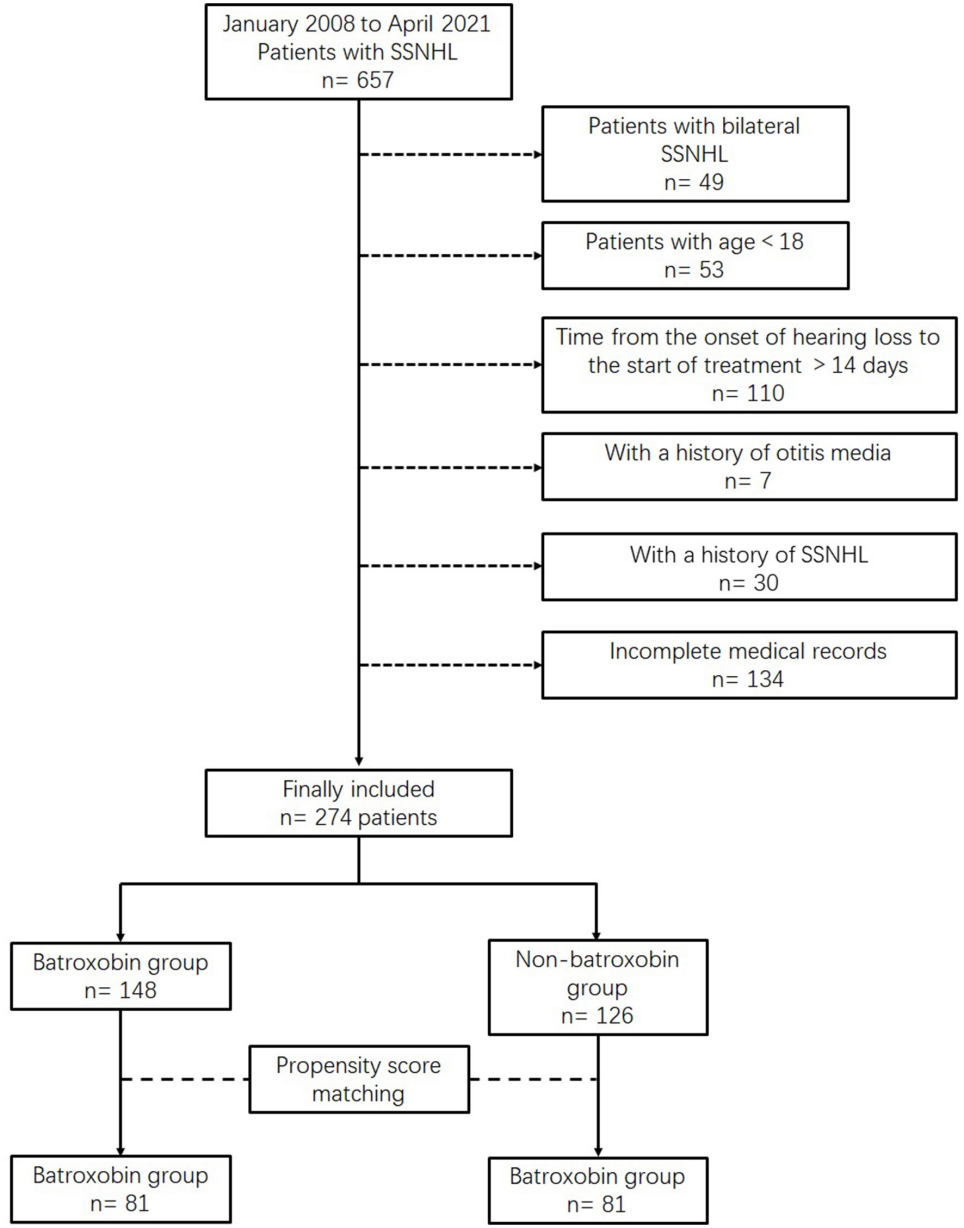
Flowchart depicting patient selection.

During the study period, 657 patients with SSNHL were admitted to our department. We excluded patients who did not meet our enrolled criteria. Finally, a total of 274 patients were included in the study: 148 for the batroxobin group and 126 for the non-batroxobin group. After PSM, a total of 162 patients (81 in each group) were included in the analysis (Figure 1). Baseline characteristics of the two groups before and after PSM are presented in Table 1. The two groups were well-balanced after PSM because all ASD values between the two groups were <0.1. The distributions of the standardized difference (SD) before and after PSM are presented in Supplementary Figure S3. After PSM, the distribution became more concentrated, being ~ 0. This indicated good balance, which was accorded with the results in Table 1. Moreover, the distribution of the propensity scores of the two groups became more similar after PSM (Supplementary Figure S1). This also exhibited that the two groups were balanced. The hospitalized treatment of SSNHL lasted for 7–10 days in our study. Once the hospitalized treatment was completed, the patients would be discharged the next day.

**Table 1:** Comparison of baseline characteristics between the batroxobin group and the non-batroxobin group in the entire cohort.

**Characteristics**	**Entire cohort (*****n*** = **274)**			**PS-matched group (*****n*** = **162)**		
	**Batroxobin group (*****n*** = **148)**	**Non-batroxobin group (*****n*** = **126)**	**ASD**	**Batroxobin group (*****n*** = **81)**	**Non-batroxobin group (*****n*** = **81)**	**ASD**
**Age, year**						
≤ 40	68 (45.9%)	70 (55.6%)		43 (53.1%)	44 (54.3%)	
>40	80 (54.1%)	56 (44.4%)	0.192	38 (46.9%)	37 (45.7%)	0.025
**Gender**						
Male	68 (45.9%)	58 (46.0%)		36 (44.4%)	38 (46.9%)	
Female	80 (54.1%)	68 (54.0%)	0.002	45 (55.6%)	43 (53.1%)	0.049
**Course of disease**						
≤ 7 days	111 (75.0%)	90 (71.4%)		62 (76.5%)	64 (79.0%)	
>7 days	37 (25.0%)	36 (28.6%)	0.082	19 (23.5%)	17 (21.0%)	0.057
**Type of pretreatment audiogram**						
Low-frequency	13 (8.8%)	24 (19.1%)		10 (12.3%)	12 (14.8%)	
High-frequency	11 (7.4%)	8 (6.3%)	0.043	3 (3.7%)	5 (6.2%)	0.097
Flat	62 (41.9%)	56 (44.4%)	0.104	39 (48.2%)	36 (44.4%)	0.074
Total-deafness	62 (41.9%)	38 (30.2%)	0.242	29 (35.8%)	28 (34.6%)	0.026
**History of hypertension**						
No	130 (87.8%)	112 (88.9%)		74 (91.4%)	72 (88.9%)	
Yes	18 (12.2%)	14 (11.1%)	0.032	7 (8.6%)	9 (11.1%)	0.075
**History of diabetes**						
No	135 (91.2%)	119 (94.4%)		76 (93.8%)	75 (92.6%)	
Yes	13 (8.8%)	7 (5.6%)	0.114	5 (6.2%)	6 (7.4%)	0.043
**Pretreatment plasma platelet count, 10** ^9^ **/L**						
≤ 200	65 (43.9%)	56 (44.4%)		40 (49.4%)	37 (45.7%)	
>200	83 (56.1%)	70 (55.6%)	0.011	41 (50.6%)	44 (54.3%)	0.074
**Pretreatment fibrinogen level, g/L**						
≤ 3	129 (87.2%)	85 (67.5%)		66 (81.5%)	65 (80.2%)	
>3	19 (12.8%)	41 (32.5%)	0.587	15 (18.5%)	16 (19.8%)	0.037
**Usage of steroids**						
Systemic alone	82 (55.4%)	112 (88.9%)		67 (82.7%)	67 (82.7%)	
Intratympanic alone	5 (3.4%)	1 (0.8%)	0.142	2 (2.5%)	1 (1.2%)	0.091
Systemic and intratympanic	61 (41.2%)	13 (10.3%)	0.471	12 (14.8%)	13 (16.1%)	0.034
**Use of HBOT**						
No	22 (14.9%)	22 (17.5%)		10 (12.3%)	11 (13.6%)	
Yes	126 (85.1%)	104 (82.5%)	0.073	71 (87.7%)	70 (86.4%)	0.035

PS, propensity score; ASD, the absolute value of the standardized mean difference; HBOT, hyperbaric oxygen therapy.

### Hearing outcomes after propensity score matching

In the propensity score-matched cohort (Table 2), according to Siegel's criteria, the complete recovery rate was 30.9% (25 of 81) for the non-batroxobin group and 24.7% (20 of 81) for the batroxobin group. Based on the CMAO criteria, the complete recovery rate was 27.2% (22 of 81) for the non-batroxobin group and 24.7% (20 of 81) for the batroxobin group. The overall effective rate was the same for Siegel's criteria and the CMAO criteria. The overall effective rate of the non-batroxobin group was 58.0% (44 of 78), whereas that of the batroxobin group was 50.6% (41 of 81). The logistic regression analysis of the propensity score-matched cohort indicated that both the complete recovery rate [Siegel's criteria, OR: 0.734, 95% CI: 0.368–1.466, *p* = 0.381; CMAO criteria, OR: 0.879, 95% CI: 0.435–1.777, *p* = 0.720] and the overall effective rates [Siegel's criteria and CMAO criteria, OR: 0.741, 95% CI: 0.399–1.378, *p* = 0.344] were of no significant difference between the two treatment groups.

**Table 2:** Complete recovery rate and overall effective rate before and after propensity score matching in the entire.

**Variables**	**Complete recovery rate**	**OR (95% CI)**	***p*-value**	**Overall effective rate**	**OR (95% CI)**	***p*-value**
**Before PSM (entire cohort)**						
**Siegel's criteria**						
Non-batroxobin group	43 of 126 (34.1%)	1	0.007	79 of 126 (62.7%)	1	0.011
Batroxobin group	29 of 148 (19.6%)	0.470 (0.272–0.814)		70 of 148 (47.3%)	0.534 (0.329–0.867)	
**CMAO criteria**						
Non-batroxobin group	38 of 126 (30.2%)	1	0.022	79 of 126 (62.7%)	1	0.011
Batroxobin group	27 of 148 (18.2%)	0.517 (0.294–0.909)		70 of 148 (47.3%)	0.534 (0.329–0.867)	
**After PSM (PS-matched cohort)**						
**Siegel's criteria**						
Non-batroxobin group	25 of 81 (30.9%)	1	0.381	47 of 81 (58.0%)	1	0.344
Batroxobin group	20 of 81 (24.7%)	0.734 (0.368–1.466)		41 of 81 (50.6%)	0.741 (0.399–1.378)	
**CMAO criteria**						
Non-batroxobin group	22 of 81 (27.2%)	1	0.720	47 of 81 (58.0%)	1	0.344
Batroxobin group	20 of 81 (24.7%)	0.879 (0.435–1.777)		41 of 81 (50.6%)	0.741 (0.399–1.378)	

PSM, propensity score matching; PS, propensity score; OR, odds ratio; CI, confidence interval; CMAO, Chinese Medical Association of Otolaryngology.

### Sensitivity analysis

**Figure 2:**
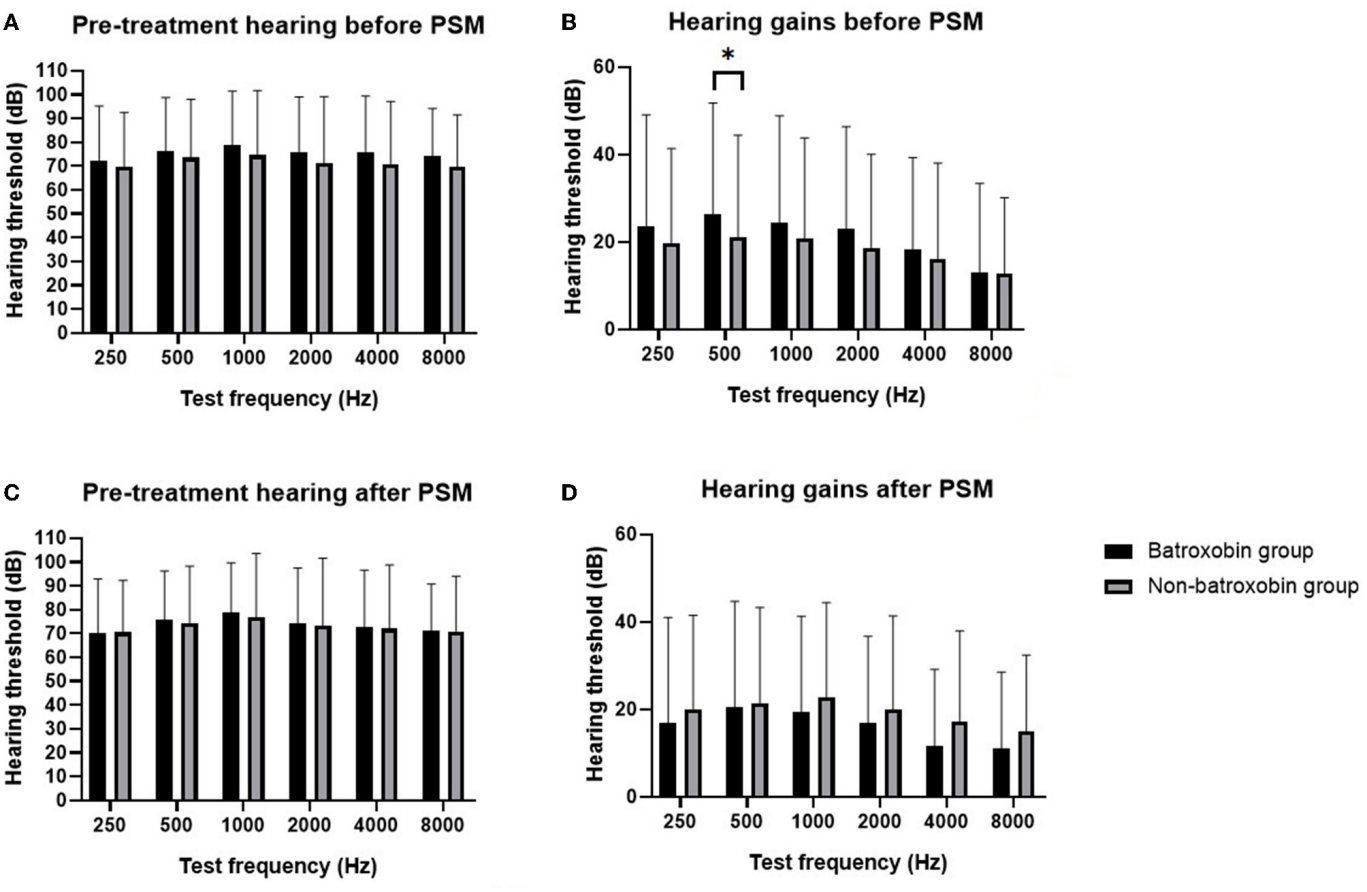
Comparison of pre-treatment hearing and hearing gains between the batroxobin group and the non-batroxobin group in the sensitivity analysis. **(A)** Pre-treatment hearing level of batroxobin group and non-batroxobin group before PSM. **(B)** Hearing gains of batroxobin group and non-batroxobin group before PSM. **(C)** Pre-treatment hearing level of batroxobin group and non-batroxobin group after PSM. **(D)** Hearing gains of batroxobin group and non-batroxobin group after PSM. The black bar represents the batroxobin group and the gray bar represents the non-batroxobin group, * stands for a *p*-value of <0.05.

In the entire cohort, 218 patients were with flat-type or total-deafness pre-treatment audiograms. PSM was used to create two groups of 61 patients each. One group was for the batroxobin group, and the other one was for the non-batroxobin group, all with flat-type or total-deafness SSNHL. These groups were well-matched in the baseline characteristics (Table 3). The distribution of SD showed that after PSM, the SD value centered at ~ 0 (Supplementary Figure S4). This implied the balance between the two groups, which was consistent with the results in Table 3. The distribution of the propensity scores of the two groups also became similar (shown in Supplementary Figure S2). As presented in Table 4, after PSM, according to Siegel's criteria, the complete recovery rate was 16.4% (10 of 61) for the non-batroxobin group and 14.8% (9 of 61) for the batroxobin group. Based on the CMAO criteria, the complete recovery rate was 13.1% (eight of 61) for the non-batroxobin group and 14.8% (nine of 61) for the batroxobin group. The overall effective rate of the non-batroxobin group was 54.1% (33 of 66) while that of the batroxobin group was 42.6% (26 of 61) for both Siegel's criteria and the CMAO criteria. The logistic regression analysis of the propensity score-matched cohort indicated that both the complete recovery rate [Siegel's criteria, OR: 0.883, 95% CI: 0.331–2.352, *p* = 0.803; CMAO criteria, OR: 1.147, 95% CI: 0.411–3.200, *p* = 0.794] and the overall effective rate [Siegel's criteria and the CMAO criteria, OR: 0.630, 95% CI 0.308–1.288, *p* = 0.206] were not significantly different between the two groups. Figure 2 shows the pre-treatment hearing levels and post-treatment hearing gains at each frequency for the two groups (the data of Figure 2 are presented in Supplementary Table S1). In the propensity score-marched cohort, there was no significant difference in the pre-treatment hearing levels and the post-treatment hearing gains between the batroxobin group and the non-batroxobin group.

**Table 3:** Comparison of baseline characteristics between the batroxobin group and the non-batroxobin group in the sensitivity analysis.

**Characteristics**	**Flat-type and total-deafness SSNHL patients (*****n*** = **218)**			**PS-matched group (*****n*** = **122)**		
	**Batroxobin group (*****n*** = **124)**	**Non-batroxobin group (*****n*** = **94)**	**ASD**	**Batroxobin group (*****n*** = **61)**	**Non-batroxobin group (*****n*** = **61)**	**ASD**
**Age, year**						
≤ 40	52 (41.9%)	46 (48.9%)		33 (54.1%)	32 (52.5%)	
>40	72 (58.1%)	48 (51.1%)	0.141	28 (45.9%)	29 (47.5%)	0.033
**Gender**						
Male	59 (47.6%)	48 (51.1%)		30 (49.2%)	32 (52.5%)	
Female	65 (52.4%)	46 (48.9%)	0.069	31 (50.8%)	29 (47.5%)	0.065
**Course of disease**						
≤ 7 days	90 (72.6%)	62 (66.0%)		44 (72.1%)	44 (72.1%)	
>7 days	34 (27.4%)	32 (34.0%)	0.148	17 (27.9%)	17 (27.9%)	0
**Type of pretreatment audiogram**						
Flat	62 (50.0%)	56 (59.6%)		34 (55.7%)	36 (59.0%)	
Total-deafness	62 (50.0%)	38 (40.4%)	0.191	27 (44.3%)	25 (41.0%)	0.065
**History of hypertension**						
No	107 (86.3%)	82 (87.2%)		54 (88.5%)	52 (85.2%)	
Yes	17 (13.7%)	12 (12.8%)	0.027	7 (11.5%)	9 (14.8%)	0.095
**History of diabetes**						
No	112 (90.3%)	88 (93.6%)		57 (93.4%)	57 (93.4%)	
Yes	12 (9.7%)	6 (6.4%)	0.111	4 (6.6%)	4 (6.6%)	0
**Pretreatment plasma platelet count, 10** ^9^ **/L**						
≤ 200	60 (48.4%)	46 (48.9%)		30 (49.2%)	31 (50.8%)	
>200	64 (51.6%)	48 (51.1%)	0.011	31 (50.8%)	30 (49.2%)	0.033
**Pretreatment fibrinogen level, g/L**						
≤ 3	108 (87.1%)	64 (68.1%)		52 (85.2%)	51 (83.6%)	
>3	16 (12.9%)	30 (31.9%)	0.533	9 (14.8%)	10 (16.4%)	0.049
**Usage of steroids**						
Systemic alone	69 (55.7%)	81 (86.1%)		49 (80.3%)	49 (80.3%)	
Intratympanic alone	4 (3.2%)	1 (1.1%)	0.149	2 (3.3%)	1 (1.6%)	0.096
Systemic and intratympanic	51 (41.1%)	12 (12.8%)	0.609	10 (16.4%)	11 (18.1%)	0.043
**Use of HBOT**						
No	17 (13.7%)	11 (11.7%)		7 (11.5%)	4 (6.6%)	
Yes	107 (86.3%)	83 (88.3%)	0.058	54 (88.5%)	57 (93.4%)	0.098

PS, propensity score; ASD, the absolute value of the standardized mean difference; HBOT, hyperbaric oxygen therapy.

**Table 4:** Complete recovery rate and overall effective rate before and after propensity score matching in the sensitivity analysis.

**Variables**	**Complete recovery rate**	**OR (95% CI)**	***p*-value**	**Overall effective rate**	**OR (95% CI)**	***p*-value**
**Before PSM (flat-type and total-deafness SSNHL patients' cohort)**						
**Siegel's criteria**						
Non-batroxobin group	15 of 94 (16.0%)	1	0.317	50 of 94 (53.2%)	1	0.127
Batroxobin group	14 of 124 (11.3%)	0.670 (0.306–1.468)		53 of 124 (42.7%)	0.657 (0.383–1.126)	
**CMAO criteria**						
Non-batroxobin group	10 of 94 (10.6%)	1	0.879	50 of 94 (53.2%)	1	0.127
Batroxobin group	13 of 124 (10.5%)	1.069 (0.453–2.526)		53 of 124 (42.7%)	0.657 (0.383–1.126)	
**After PSM (PS-matched cohort)**						
**Siegel's criteria**						
Non-batroxobin group	10 of 61 (16.4%)	1	0.803	33 of 61 (54.1%)	1	0.206
Batroxobin group	9 of 61 (14.8%)	0.883 (0.331–2.352)		26 of 61 (42.6%)	0.630 (0.308–1.288)	
**CMAO criteria**						
Non-batroxobin group	8 of 61 (13.1%)	1	0.794	33 of 61 (54.1%)	1	0.206
Batroxobin group	9 of 61 (14.8%)	1.147 (0.411–3.200)		26 of 61 (42.6%)	0.630 (0.308–1.288)	

PSM, propensity score matching; PS, propensity score; OR, odds ratio; CI, confidence interval; CMAO, Chinese Medical Association of Otolaryngology.

## Discussion

Sudden sensorineural hearing loss is an urgent situation for the otolaryngology department. Patients are usually concerned about permanent hearing loss. Meanwhile, losing the sense of hearing can cause great inconvenience for patients. Impaired cochlea microcirculation was proposed to be a pathogenic factor (15–17). SSNHL is also associated with an increased risk of stroke and myocardial infarction (18–20). Batroxobin, which can increase cochlear blood flow (21), was suggested to be applied in the treatment of SSNHL (2), especially for flat-type and total-deafness SSNHL (12, 22). We used both Siegel's criteria and the CMAO criteria to grade hearing recovery. However, our results indicated that no matter the complete recovery rates or the overall effective rates were with no significant difference between the batroxobin group and the non-batroxobin group in the entire cohort after PSM by both criteria. For patients with flat-type and total-deafness SSNHL, the complete recovery rates and the overall effective rates by the two criteria were still without significant differences between the two treatment groups after PSM. Moreover, no meaningful difference was found between the two treatment groups in the post-treatment hearing gains at all frequencies after PSM.

Studies have demonstrated that no obvious difference was found in fibrinogen level (23, 24), activated partial thromboplastin time, and prothrombin time (25) between SSNHL patients and normal people. The hearing recovery of SSNHL patients was not related to the fibrinogen level (26). These findings may lead to negative results in our study. Older age, a longer course of disease (27, 28), total-deafness type of pre-treatment audiogram (29), and history of diabetes (30) have been considered adverse factors of hearing recovery for SSNHL patients. The use of steroids or HBOT (31) can also affect hearing outcomes. Our study has used PSM to balance the abovementioned potential confounders. Meanwhile, we also adjusted other underlining confounders such as gender, history of hypertension, and pre-treatment plasma platelet count. Before PSM, the complete recovery rates and the overall effective rates of the batroxobin group were significantly lower in the entire cohort (Table 2). According to Table 1, patients in the batroxobin group were older, and more patients suffering from diabetes and total deafness type of SSNHL. After using PSM to balance these possible confounders, the complete recovery rates and the overall effective rates became similar between the two groups (Table 2). Before PSM, hearing gain at 500 Hz of the batroxobin group was significantly higher in the flat-type and total-deafness SSNHL patients (Figure 2). However, patients in the batroxobin group were often treated together with intratympanic steroids or intratympanic and systematic steroids, while patients in the non-batroxobin group were often treated with systematic steroids alone (Table 3). It has been demonstrated that intratympanic steroids were better for PTA improvement than systematic steroids (32). Thus, the different applications of steroids between the two groups could be a confounding factor to the results. After PSM, the difference in hearing gains between the two groups became statistically unmeaningful (Figure 2). These indicated the importance and the advantage of using PSM. Kubo et al. once proposed that defibrinogenation therapy can achieve better hearing outcomes than steroid therapy (33). However, he did not rule out confounding factors such as age, and audiogram patterns. A randomized controlled trial in China (34) showed that batroxobin + Ginaton + corticosteroid had the highest efficiency in the treatment of total-deafness SSNHL patients, compared with batroxobin alone, batroxobin + Ginaton, and Ginaton + corticosteroid. However, among the four treatment groups, the two groups treated with corticosteroid were both with higher recovery rates, and the other two groups treated with batroxobin did not do so well. Therefore, better hearing outcomes in the trial may mainly result from the use of corticosteroids instead of batroxobin. In addition, a clinical trial has not evaluated hearing gains at each frequency for total-deafness SSNHL patients. The possible complications of batroxobin treatment should also be taken into account. Dizziness, nausea, and purpura on the skin have been reported in patients after treatment with batroxobin (13). There could be risks for hemorrhagic tendency as well.

To the best of our knowledge, our study was the first one that used PSM to analyze the effect of batroxobin in the treatment of SSNHL. PSM can partly eliminate the discrepancies between two cohorts of patients. After PSM, the baseline information becomes compatible. Using PSM can help researchers to avoid potential bias (35). The use of batroxobin in the treatment of SSNHL was controversial. We have added more evidence for medication options when treating patients with SSNHL.

There were still several limitations to this study. First of all, the number of cases was limited. The patients admitted were only in one single institution, which limited the generalization of our findings to other populations. Moreover, because of its retrospective design, there could be selection biases. Finally, we only assessed the hearing improvement right after therapy. As for the long-term hearing outcomes and complications after treatment, future studies are still needed.

## Conclusion

There was no significant difference in short-term hearing outcomes between treatment combined with batroxobin and treatment without batroxobin in SSNHL patients by both Siegel's criteria and the CMAO criteria after PSM. Future studies for better therapy regimens of SSNHL are still needed.

## Data availability statement

The raw data supporting the conclusions of this article will be made available by the authors, without undue reservation.

## Ethics statement

The studies involving human participants were reviewed and approved by the Ethical Committee of Xiangya Hospital, Central South University. The Ethics Committee waived the requirement of written informed consent for participation.

## Author contributions

XWu, MJ, and HH: study design, data analysis and interpretation, and draft writing. XWu, MJ, HH, HW, and XWa: data collection. LM, CH, XC, LJ, and HW: technical and material support. All authors have read and approved the final manuscript.

## Supplementary material

Supplementary Figure S1 Distribution of propensity scores before and after propensity score matching in the entire cohort. Treated as the batroxobin group, control as the non-batroxobin group.

Supplementary Figure S2 Distribution of propensity scores before and after propensity score matching in flat-type and total-deafness SSNHL patients. Treated as the batroxobin group, control as the non-batroxobin group.

Supplementary Figure S3 Distribution of standardized difference before and after propensity score matching in the entire cohort.

Supplementary Figure S4 Distribution of standardized difference before and after propensity score matching in flat-type and total-deafness SSNHL patients.

Supplementary Table S1 Comparison of pre-treatment hearing and hearing gains between the batroxobin group and the non-batroxobin group in the sensitivity analysis.
