# Supplementary figures and images for: Short-term effects of intravenous batroxobin in treatment of sudden sensorineural hearing loss: a propensity score-matched study
Source: Front Neurol. 2023 Apr 17;14:1102297. doi: 10.3389/fneur.2023.1102297 (PMC10150045; doi:10.3389/fneur.2023.1102297)

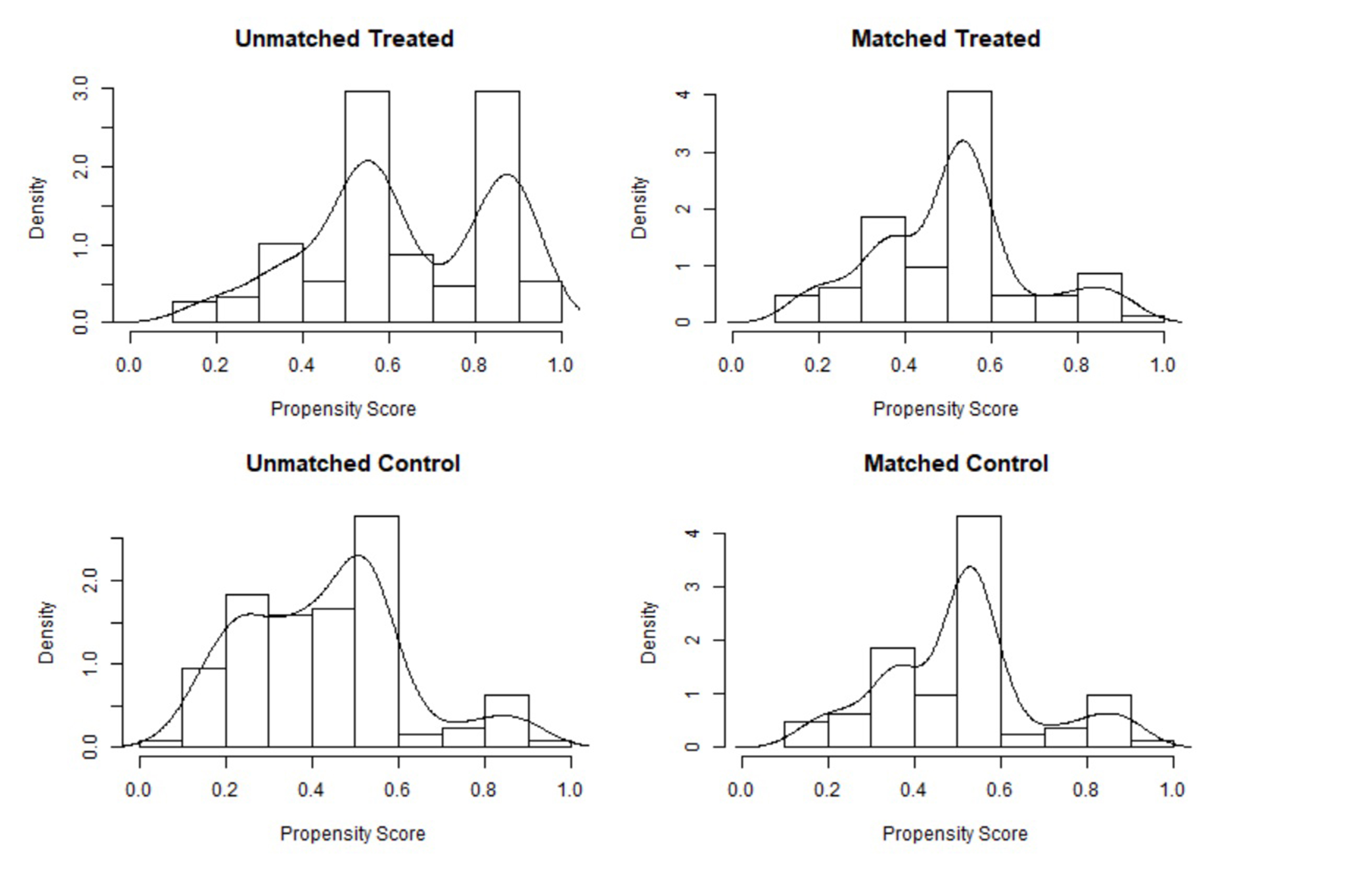

Supplement: Supplementary Figure S1 — Distribution of propensity scores before and after propensity score matching in the entire cohort. Treated as the batroxobin group, control as the non-batroxobin group. [file Image_1.JPEG]

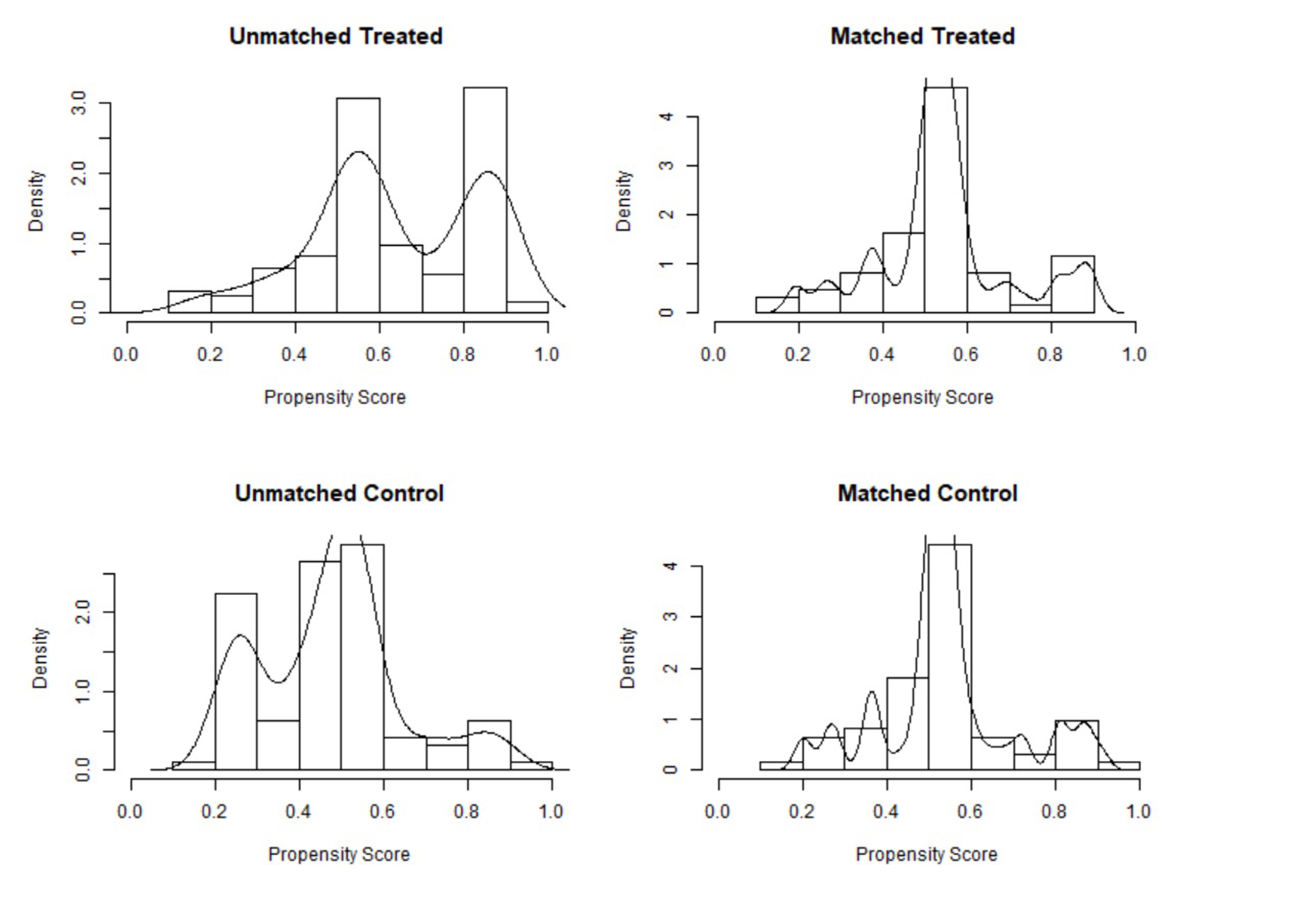

Supplement: Supplementary Figure S2 — Distribution of propensity scores before and after propensity score matching in flat-type and total-deafness SSNHL patients. Treated as the batroxobin group, control as the non-batroxobin group. [file Image_2.JPEG]

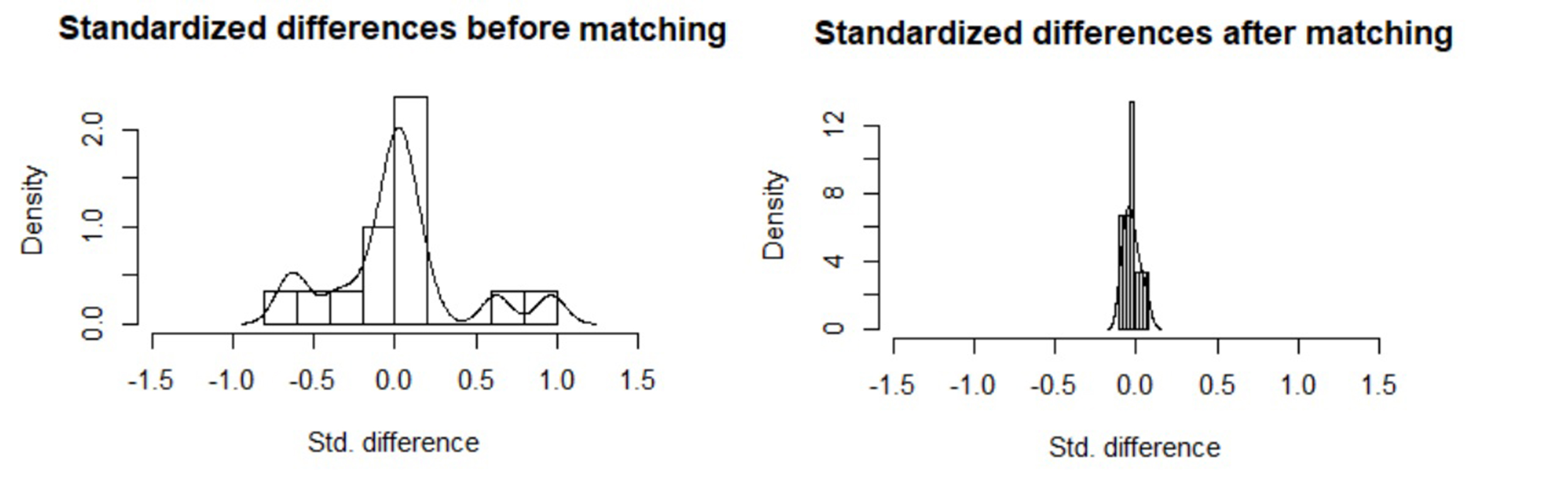

Supplement: Supplementary Figure S3 — Distribution of standardized difference before and after propensity score matching in the entire cohort. [file Image_3.JPEG]

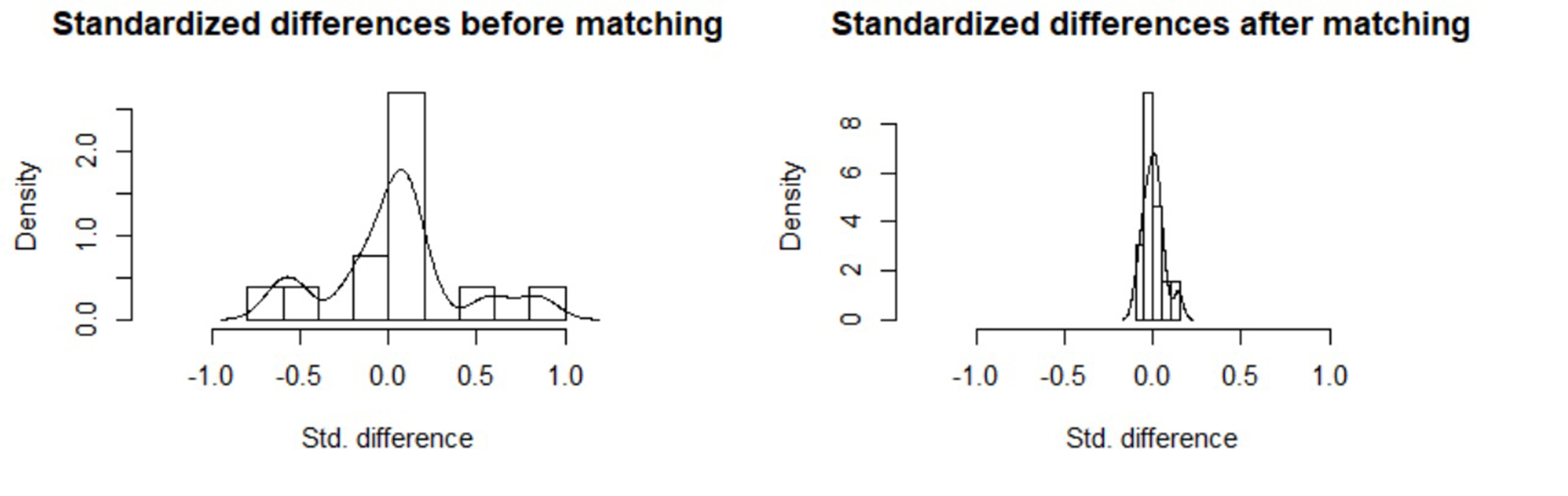

Supplement: Supplementary Figure S4 — Distribution of standardized difference before and after propensity score matching in flat-type and total-deafness SSNHL patients. [file Image_4.JPEG]
