# Supplementary material for: Short-term effects of intravenous batroxobin in treatment of sudden sensorineural hearing loss: a propensity score-matched study
Source: Front Neurol. 2023 Apr 17;14:1102297. doi: 10.3389/fneur.2023.1102297 (PMC10150045; doi:10.3389/fneur.2023.1102297)
Supplement: Supplementary Table S1 — Comparison of pre-treatment hearing and hearing gains between the batroxobin group and the non-batroxobin group in the sensitivity analysis. [file Table_1.DOCX]

**Table S1 Comparison of Pre-treatment Hearing and Hearing Gains between Batroxobin Group and Non-batroxobin Group in the Sensitivity analysis**

|  | **Batroxobin Group** | | **Non-batroxobin Group** | **P-value** |
| --- | --- | --- | --- | --- |
| **Before PSM (flat-type and total-deafness SSNHL patients’ cohort)** | | | | |
| Pre-treatment hearing at 250Hz | | 72.07±23.12 | 69.84±22.61 | 0.487 |
| Pre-treatment hearing at 500Hz | | 76.44±22.31 | 73.56±24.39 | 0.362 |
| Pre-treatment hearing at 1000Hz | | 78.62±22.69 | 74.57±27.01 | 0.215 |
| Pre-treatment hearing at 2000Hz | | 75.58±23.13 | 71.30±27.81 | 0.197 |
| Pre-treatment hearing at 4000Hz | | 75.90±23.44 | 70.90±26.25 | 0.107 |
| Pre-treatment hearing at 8000Hz | | 74.10±20.05 | 69.63±21.88 | 0.097 |
|  |  | |  |  |
| Hearing Gain at 250Hz | 23.51±25.57 | | 19.79±21.54 | 0.171 |
| Hearing Gain at 500Hz | 26.22±25.56 | | 21.08±23.35 | 0.037 |
| Hearing Gain at 1000Hz | 24.26±24.60 | | 20.74±23.04 | 0.181 |
| Hearing Gain at 2000Hz | 22.87±23.49 | | 18.62±21.39 | 0.061 |
| Hearing Gain at 4000Hz | 18.24±21.02 | | 16.17±21.84 | 0.368 |
| Hearing Gain at 8000Hz | 12.98±20.38 | | 12.66±17.46 | 0.876 |
|  | | | | |
| **After PSM (PS-matched cohort)** | | | | |
| Pre-treatment hearing at 250Hz | | 70.00±22.86 | 70.49±21.75 | 0.900 |
| Pre-treatment hearing at 500Hz | | 75.57±20.66 | 74.51±23.71 | 0.764 |
| Pre-treatment hearing at 1000Hz | | 78.77±20.81 | 76.72±26.80 | 0.594 |
| Pre-treatment hearing at 2000Hz | | 74.18±23.17 | 73.39±28.13 | 0.831 |
| Pre-treatment hearing at 4000Hz | | 72.95±23.60 | 71.97±26.70 | 0.786 |
| Pre-treatment hearing at 8000Hz | | 71.31±19.43 | 70.90±23.07 | 0.908 |
|  |  | |  |  |
| Hearing Gain at 250Hz | 16.80±24.17 | | 20.08±21.44 | 0.446 |
| Hearing Gain at 500Hz | 20.41±24.26 | | 21.34±21.91 | 0.826 |
| Hearing Gain at 1000Hz | 19.34±21.94 | | 22.70±21.67 | 0.381 |
| Hearing Gain at 2000Hz | 16.80±19.85 | | 19.95±21.43 | 0.380 |
| Hearing Gain at 4000Hz | 11.64±17.50 | | 17.21±20.69 | 0.115 |
| Hearing Gain at 8000Hz | 11.10±17.43 | | 14.92±17.45 | 0.347 |

Abbreviations: PSM, propensity score matching; PS, propensity score.
